# Right Ventricular Function Improves After Bench Press: A Speckle Tracking Echocardiography Study

**DOI:** 10.3390/medicina61081469

**Published:** 2025-08-15

**Authors:** María Belén Martínez-Lechuga, Javier Hidalgo-Martín, Manuel Ruiz-Bailén

**Affiliations:** 1 Jaén University Hospital, 23007 Jaén, Spain; mariab.martinez.sspa@juntadeandalucia.es; 2 Cardiac Care Unit, Intensive Care Unit, Jaén University Hospital, 23007 Jaén, Spain; jhidalgomartin1@gmail.com; 3 Department of Health Sciences, Faculty of Health Sciences, University of Jaén, Campus Las Lagunillas, 23071 Jaén, Spain

**Keywords:** speckle tracking, 3D echocardiography, athlete, bench press, right ventricle

## Abstract

*Background and Objectives*: The association between right ventricular myocardial fiber deformation and nutrition in weightlifters has not been fully characterized. This study analyzed nutritional factors and right ventricle speckle tracking echocardiography parameters in weightlifters before and after bench press exercises. *Materials and Methods*: This interventional study examined the effects of bench press exercises on myocardial function. Nutritional parameters were assessed prior to exercise. Echocardiography with speckle tracking using vector velocity analysis was performed before and immediately after the bench press exercise. This study included a group of non-elite athlete weightlifters and a non-athlete control group to compare right myocardial function. In the athlete group, transthoracic echocardiograms (TTEs) were conducted before and after the exercise to assess changes in systolic and diastolic right heart function. A cohort of 30 weightlifters from 2014 who continued regular training was re-evaluated in 2024, and nutritional data were collected. Data analyses included ANOVA and Student’s T-tests, and correlation coefficients were calculated to explore associations with speckle tracking results. *Results*: This study involved 211 male weightlifters and a control group of 60 non-athletes. Measured values for the control group and athletes before and after bench press exercise were as follows: right longitudinal global strain (−27.31 ± 1.47, −23.55 ± 2.37, −30.98 ± 2.12); right global longitudinal strain rate (−1.79 ± 0.078, −1.48 ± 0.33, −2.88 ± 0.259 1/s), all *p* < 0.001; and isovolumic acceleration (2.38 ± 0.22, 3.52 ± 0.15, 6.66 ± 0.88 m/s^2^, *p* < 0.001). Following exercise, right intraventricular synchrony increased, and longitudinal strain delay decreased (144.88 ± 22.52, 168.92 ± 29.35, 98.27 ± 12.11 ms, *p* < 0.001). The follow-up group demonstrated a similar response to exercise as the other weightlifters. Right ventricular longitudinal strain showed correlations with protein, vitamin E, and zinc levels (R^2^ = 0.399, *p* = 0.021; R^2^ = 0.378, *p* = 0.03; R^2^ = 0.566, *p* < 0.01), and right ventricular radial velocities correlated with group B vitamins. *Conclusions*: Weightlifters show less right ventricular deformity before exercise compared to controls, but their strain increases significantly post-exercise. Speckle tracking values might correlate with nutrition.

## 1. Introduction

Cardiovascular physiology, especially new insights from cardiac resonance and speckle tracking echocardiography, is a fascinating and rapidly advancing field of study. However, findings vary due to differences in technique, sport type, training specifics, duration, nutrition, sex, race, age, and genetics [1,2,3,4,5,6,7,8,9,10,11,12,13,14,15,16,17,18,19].

Studies have shown that ultramarathon running could reduce left ventricular ejection fraction (LVEF) and strain [2], or induce deleterious effects [1,7,8,9,10], whereas cycling may not impair or worsen right ventricular function [11] or might even improve the diastolic function of the left ventricle (LV) [12]. Furthermore, training in soldiers can lead to asymmetric hypertrophy [13] or dilation of the heart chambers without resulting in functional changes [11]. In addition, the cardiovascular adaptations observed in judokas [14,15,16], other martial artists [15], and individuals undergoing military training [13,14,17,18] demonstrate the specific and varied remodeling capabilities in response to different physical disciplines [19].

Research has predominantly focused on left ventricular function, but the right ventricle (RV) is also important during and after intense physical activity [1]. Assessing RV function can be more challenging. Cardiac resonance and echocardiography have shown structural changes due to exercise-induced volume overload [9,12,13]. In this regard, Aaron et al. [20] demonstrated that RV mass and RV end-diastolic volumes increased with the level of physical activity, independent of LV measures. Differential stress in endurance athletes produced in both ventricles could disproportionately affect the RV, inducing acute changes in RV structure and function that are more prevalent and more profound than for the left ventricle, and can even cause elevations in pulmonary artery pressure. These findings suggest that prolonged and intense physical activity could cause cardiac fatigue, especially in the RV [7,18,20,21], potentially leading to severe arrhythmias [22,23].

Information about right ventricular function in athletes compared to non-athletes varies, indicating that it may be normal, enhanced, or reduced. This suggests that multiple factors influence remodeling, and further research is urgently needed to gain a better understanding of the issue [1,8,11,24,25,26].

On the other hand, it has been shown that nutritional status [27], including vitamin levels [5], influences myocardial performance, which can be demonstrated by the response of myocardial fibers as measured by speckle tracking values. However, the relationship between the nutritional profile of strength athletes and their cardiac remodeling has currently remained elusive.

Studies are typically conducted on elite athletes in selected disciplines such as swimming [5], cycling [11,12], and running [1,3,9,10]. In comparison, weightlifting is a discipline that has seen a rise in participation over recent years, but studies using the speckle tracking technique are lacking [28,29]. This echocardiographic method provides detailed information about the physiological aspects associated with physical activities [30].

Hence, here we aim to assess right ventricular function differences between untrained and trained weightlifters, including changes after bench press exercise. Specifically, we seek to measure speckle tracking parameters like strain, strain rate, velocities, and displacement at baseline and post-exercise. Additionally, we will evaluate how nutritional factors relate to these cardiac measurements.

## 2. Materials and Methods

### 2.1. Study Design

Measurements were taken before and immediately after exercise. Echocardiography was conducted on the bench press apparatus. Initially, echocardiography was performed, followed by the bench press exercise, and immediately afterward, another echocardiography was conducted. The inclusion phase occurred from June 2014 to February 2020, and from 2023 to December 2024. The control group was included from 2015 to April 2025. This study was paused during the years 2020 to 2022 due to the COVID-19 pandemic. This research was performed as part of the PAIDI CTS 606 Andalusian Health Service Project No. PI-0585-2012. It has received approval from the local ethics committee and funding from the Department of Health of the Government of Andalusia, Spain.

### 2.2. Follow-Up

This longitudinal study began in 2014. Weightlifting athletes were examined, and 30 athletes who continued training had an additional follow-up echocardiography after 10 years.

### 2.3. Candidates Included in This Study

#### Control Group

**Figure 1:**
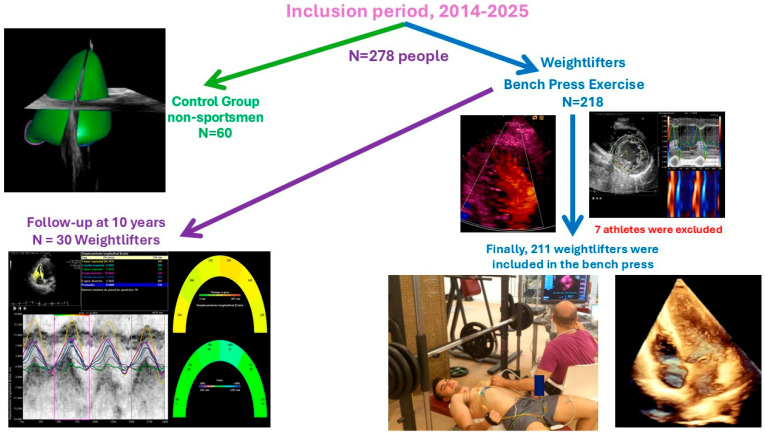
Population included in the study. Permission was granted from the people appearing in the image.

The control group included 60 healthy, untrained individuals who did not engage in bench pressing or other strength exercises. The control group underwent transthoracic echocardiography (TTE) with advanced imaging at the Coronary Unit of the “Hospital Universitario de Jaén, Spain”, following identical procedures as the athletes. This group was divided into two subgroups of 30 individuals each: one to compare with the weightlifters who did not participate in a follow-up evaluation and the other to compare with the follow-up group. This ensures that the ages of the control group and the investigated groups are similar. The control group was created to establish reference echocardiographic values (Figure 1. Table 1, Table 2, Table 3, Table 4 and Table 5).

### 2.4. Weightlifters Participating in the Stress Test

We studied Caucasian male weightlifters aged 25–45 who regularly train with the bench press. Athletes are defined as engaging in over 3000 MET-minutes of physical activity per week, measured by the Spanish version of the IPAQ (2002). See Figure 1.

The following candidates were excluded from this study: 1. Athletes with any known cardiovascular disease previously known or detected during the study. 2. Athletes with poor echocardiography quality in their video recording. 3. Individuals with a previous diagnosis of arrhythmias. 4. Individuals with a previous diagnosis of hypertension.

#### 2.4.1. Intervention: Study on the Cohort

All included candidates underwent echocardiography. TTE was recorded in high-quality digital format. The analysis was performed offline and blindly using the “Syngo software program, U.S. Siemens, 2013^®^ (Mountain View, CA, USA)”. Three independent studies were conducted to evaluate the extent of inter-observer agreement. Biochemistry and hemogram tests were performed on 67 volunteer athletes who consented to have their blood samples taken 8 h post-exercise. In these tests, the following parameters were assessed: troponin I, brain natriuretic peptide, interleukin 6, poly-unsaturated fatty acids (PUFA), sodium, potassium, vitamins B, C and D, folic acid, magnesium, zinc, C-reactive protein, interleukin 6, and creatinine.

#### 2.4.2. Intervention: Exercise Stress-Echo in Weightlifters’ Group

Measurements were taken before and after exercise. Echocardiography was performed immediately prior to strength training. Following the echocardiography, the bench press exercise was performed. Subsequently, a new echocardiography was performed immediately after the exercise. The degree of strength training was assessed by the maximum load for 1 repetition maximum (RM). This test was performed at least three days before the bench press study to prevent muscle fatigue. To evaluate the strength of the chest muscles and prevent injuries, the following protocol was provided to athletes: 1. Start with a warm-up set with a light load allowing you to perform 10 repetitions. 2. Rest for one minute. 3. Perform another series with a resistance heating that will allow complete 3–5 repetitions. (This usually meant an increase of 5–10% of the previous set.) 4. Rest for two minutes. 5. Estimate another increase (5–10%) that allows you to fully 2 or 3 repetitions. 6. Rest for four minutes. 7. Estimate another increase (5–10%) that allows for a single repetition of the exercise, carried out correctly. 8. Rest four minutes and then calculate a new moderate increase in weight (5–10%) and repeat the test. 9. If you cannot lift the weight, after the four-minute break, the weight (2.5 to 5%) will be reduced, and then repeat. Continue increasing or decreasing the weight as necessary to determine their repetition maximum (RM) real. After determining the training load using the 1 RM test, the load can move a total of 10 replicates were estimated. The training protocol was based on the 10 sets of 10 repetitions with a current load of 75% of 1 RM estimating, each with a break of 1′3′′ between sets. After completing the exercise, series, and rests, echocardiography was performed.

#### 2.4.3. Image Acquisition and Processing

**Figure 2:**
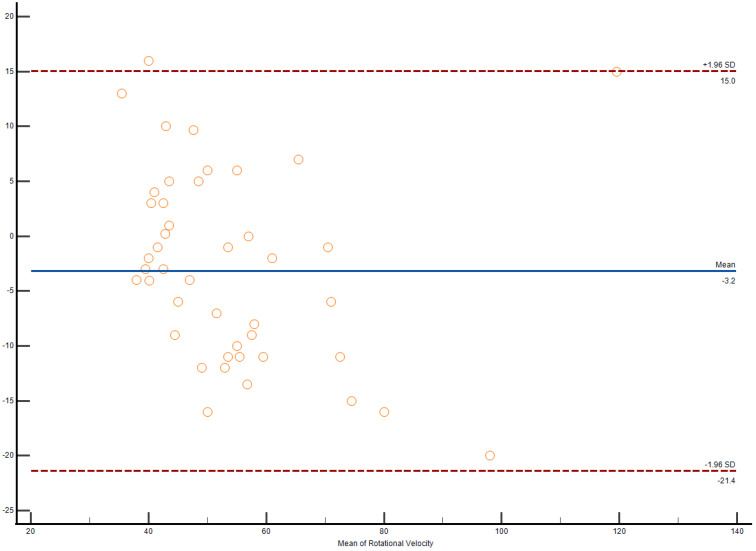
Bland–Altman plot demonstrates interobserver agreement in longitudinal strain measurements of the right ventricle among weightlifters.

**Figure 3:**
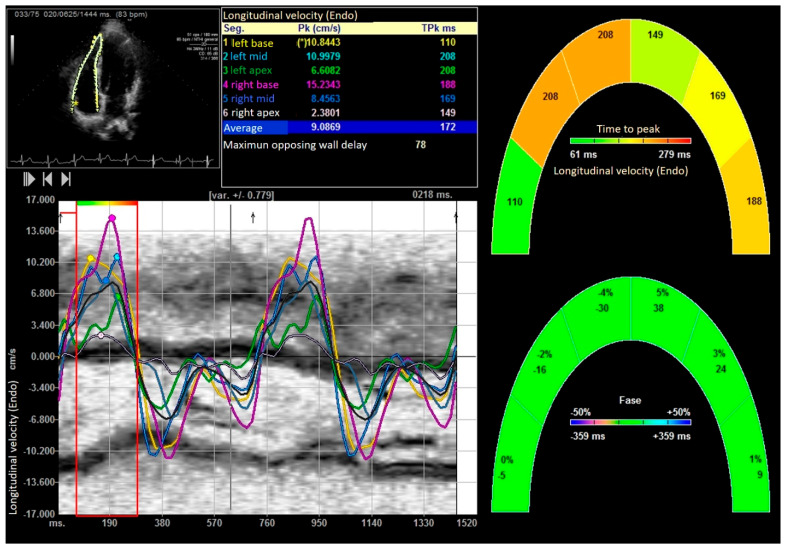
Longitudinal velocity of right ventricle. “*” indicates the laterobasal segment of the right ventricle.

**Figure 4:**
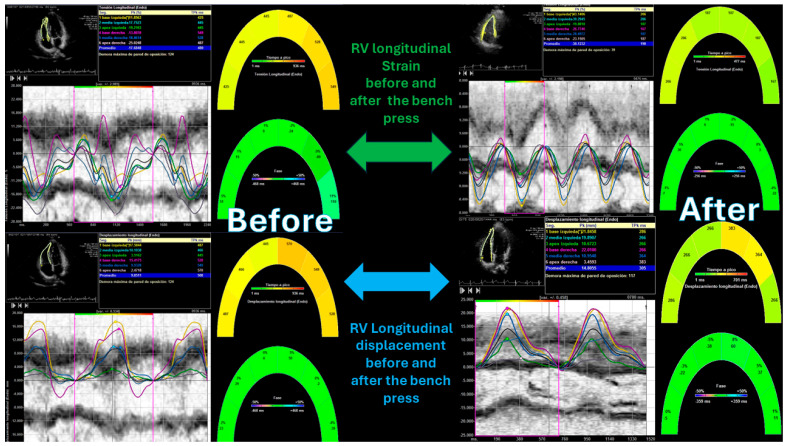
Right ventricular strain and longitudinal displacement before and after the bench press exercise. “*” indicates the laterobasal segment of the right ventricle.

**Figure 5:**
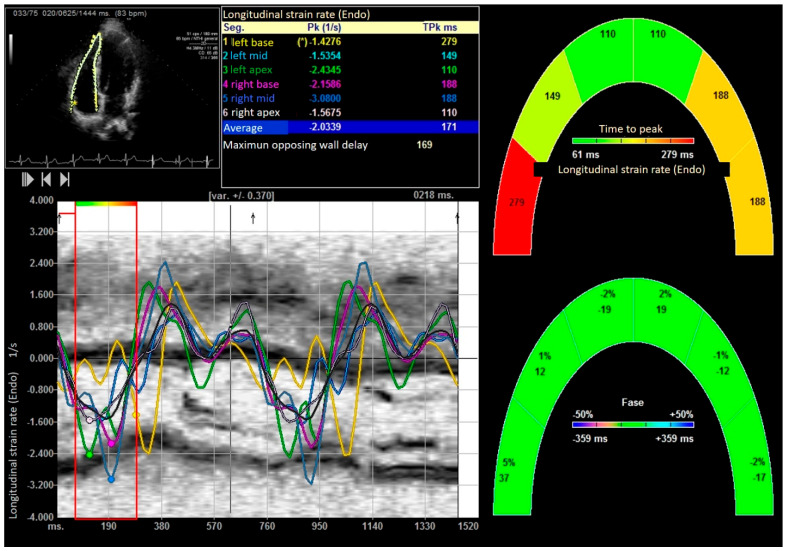
Global longitudinal strain rate of right ventricle. “*” indicates the laterobasal segment of the right ventricle.

Standard transthoracic echocardiograms were performed using commercially available systems (SC 2000 from Siemens^®^ (Mountain View, USA) in the U.S. and Sequoia 512c (Mountain View, CA, USA)). The examinations were conducted in a decubitus supine position. Apical four-chamber orientation was used to acquire right ventricular functional data. A six-segment model was adopted to assess regional and global right ventricular performance in the longitudinal direction. The region of interest was manually traced along the endomyocardial border in the six segments. The frame rates ranged from 70 to 120 frames per second, utilizing multiple focal points. All images were optimized with adjustments for gain, compression, and dynamic range. Probes 4V1C and 4Z1c were used. Off-line analysis was performed blindly using Syngo software by Siemens^®^ (2013) (Mountain View, USA). Usual echocardiographic parameters recommended by the American Society of Echocardiography were evaluated [31,32]. Right ventricular ejection fraction (RVEF) was calculated using both 2D and 3D images [33]. Right ventricular systolic function was also evaluated using right ventricular isovolumic acceleration (IVA) [31,32,34,35]. The quantification of the right E/E′ ratio and parameters derived from speckle tracking, including strain, strain rate, displacement, as well as longitudinal and radial velocities of the right ventricle, were evaluated. Figure 2, Figure 3, Figure 4 and Figure 5. Global right ventricular strain, including the lateral wall and interventricular septum, was assessed. The right ventricle was divided into six segments in the apical four-chamber view: laterobasal, medial lateral, lateroapical, septoapical, septal medial, and septobasal. The shortening fraction was measured in each segment before and after strength training.

#### 2.4.4. Statistical Analysis

ANOVA and Student’s *t*-test assessed differences in quantitative variables after checking normality and homoscedasticity. Paired samples ANOVA compared control group, pre-exercise weightlifters, and post-exercise weightlifters. Student’s *t*-test evaluated age differences within the control group and weightlifters. This test was also used to assess right ventricular remodeling in the cohort of 30 athletes who were followed up for 10 years. Nonparametric tests, especially the Kruskal-Wallis test, were used for variables that did not follow a normal distribution. Multiple correlations among echocardiographic variables, the load applied in 1 RM, and nutritional data were performed using Pearson’s correlation coefficient or Spearman’s test, based on variable distribution. Absolute values of the variables obtained from speckle tracking were utilized. The degree of interobserver agreement for right longitudinal strain and strain rate was assessed by Bland–Altman plots. Results were presented as means and standard deviations, with a *p*-value < 0.05 considered statistically significant. The IBM SPSS version 29 software, Chicago, IL, USA, was employed for all statistical analyses.

## 3. Results

### 3.1. Study Participants

Initially, 218 Caucasian weightlifters were included in this study, as shown in Figure 1. Seven athletes did not complete it. Among them, one had noncompaction cardiomyopathy, three were excluded for syncope, one for atrial fibrillation as a result of Wolff–Parkinson–White syndrome, another experienced non-sustained ventricular tachycardia due to arrhythmogenic right ventricular cardiomyopathy and is awaiting a heart transplant, and one could not finish the exercise. Finally, 211 athletes completed the study with a mean RM of 88.54 ± 16.33 kg. Additionally, 30 non-athletes were included as a control group with an average age of 33.47 ± 9.21 and 29.33 ± 14.24 years (*p* = 0.052, T-students test), as shown in Figure 1.

### 3.2. Physiological Parameters

Despite the statistically significant hemodynamic differences observed, these did not translate into clinical relevance. Post-exercise, both blood pressure and heart rate showed a moderate increase, with weightlifters demonstrating a lower baseline heart rate compared to the control group. Table 1.

**Table 1:** Comparative analysis of athletes’ physiological parameters before and after bench press, and control groups, using ANOVA and Student’s *t*-test.

	Control Group (*n* = 60)	Before Bench Press (*n* = 211)	After Bench Press (*n* = 211)	*p*-Value
Weight (Kg)	82.43 ± 13.28	81.77 ± 11.44		0.173
Height (m)	1.77 ± 15.27	1.78 ± 23.54		0.540
Body Surface (Dubois and Dubois m^2^)	2.014 ± 0.32	2.001 ± 0.17		0.128
Systolic blood pressure (mmHg)	119.41 ± 18.75	122.38 ± 13.27	135.87 ± 15.48	0.001
Diastolic blood pressure (mmHg)	81.28 ± 22.32	65.27 ± 16.32	85.88 ± 25.48	0.001
Heart rate B/min	74.35 ± 12.10	58.75 ± 11.32	101.58 ± 24.44	0.001
Breathing frequency (B/m)	16.22 ± 3.54	14.22 ± 9.22	24.32 ± 5.2	0.001
SpO_2_	97.12 ± 0.88	95.02 ± 0.12	94.78 ± 2.32	0.45

### 3.3. Echocardiography Parameters

The Bland–Altman plot demonstrates interobserver agreement in longitudinal strain measurements of the right ventricle among weightlifters, as shown in Figure 2.

### 3.4. Right Ventricular Systolic Function

Right ventricular wall thickness in the subcostal window was 5.11 ± 0.08 cm in weightlifters versus 4.16 ± 0.32 mm in control group, *p* value > 0.05. The control group had a higher right ejection fraction on 2D and 3D echocardiography and a higher TAPSE value, but lower tissue velocities (both S’ wave and IVA) than the athletes at baseline. After exercise, the ejection fraction and all parameters of systolic function increased. Basal segments of weightlifters show a smaller segmental shortening fraction than overall (0.49 ± 0.06 versus 0.43 ± 0.02, *p* value 0.032) by VVI analysis, as shown in Table 2 and Figure 1.

**Table 2:** Right ventricular systolic function. Anova test was used.

	Control Group *(n* = 60)	Before Bench Press (*n* = 211)	After Bench Press (*n* = 211)	*p*-Value
3D RVEF (%)	0.67 ± 0.06	0.55 ± 0.22	0.78 ± 0.12	0.001
IVA (m/s^2^)	2.38 ± 0.22	3.52 ± 0.15	6.66 ± 0.88	0.001
S’ wave (cm/s)	12.27 ± 1.13	14.52 ± 0.09	18.38 ± 1.21	0.001
TAPSE (mm)	24.33 ± 3.12	19.08 ± 5.14	27.45 ± 3.21	0.001
EDRVD (mm)	37.22 ± 0.11	42.13 ± 0.08	48.24 ± 0.92	0.001
Global RVEF 2D	0.62 ± 0.15	0.49 ± 0.06	0.74 ± 3.21	0.001
Laterobasal RVEF	0.65 ± 0.25	0.42 ± 0.02	0.94 ± 0.17	0.001
Lateromedial RVEF	0.62 ± 0.12	0.55 ± 0.18	0.74 ± 0.22	0.001
Lateroapical RVEF	0.62 ± 0.09	0.49 ± 0.17	0.95 ± 0.19	0.001
Septoapical RVEF	0.63 ± 0.15	0.47 ± 0.18	0.95 ± 0.21	0.001
Septomedial RVEF	0.65 ± 0.21	0.46± 0.22	0.98 ± 0.17	0.001
Septobasal RVEF	0.58 ± 0.22	0.43 ± 0.02	0.94 ± 0.19	0.001

IVA: Isovolumic acceleration (m/s). Global RVEF: right ventricular ejection fraction. EDRVD: end-diastolic right ventricular diameter, evaluated in apical 4-chamber projection, measured at the base of the right ventricle.

**Figure 6:**
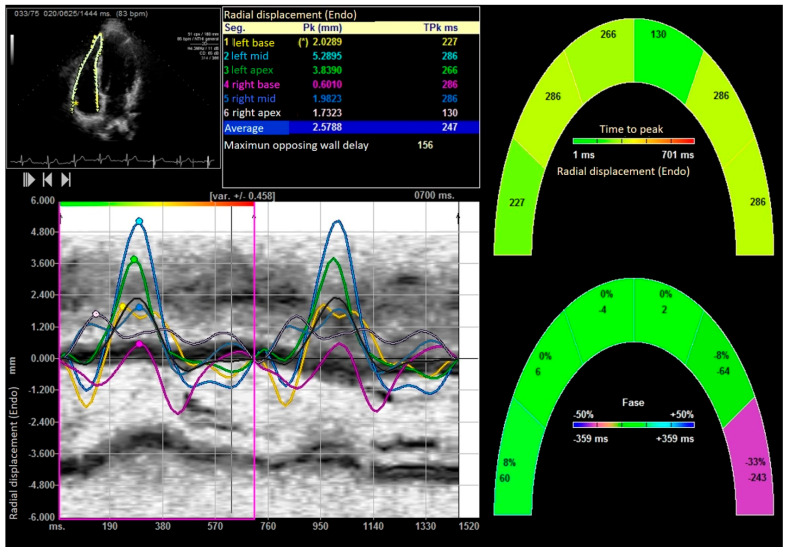
Right ventricular radial displacement. “*” indicates the laterobasal segment of the right ventricle.

**Figure 7:**
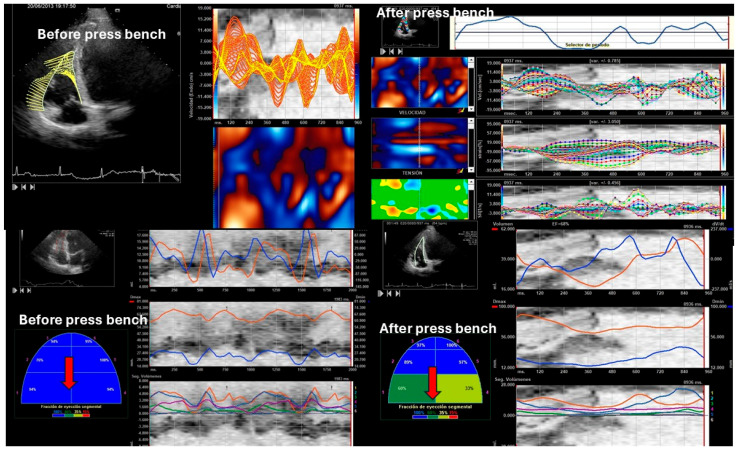
Speckle tracking before and after bench pressing. The red arrows point to the basal segments, and a deterioration of segmental contractility of the basal segments can be observed by hybrid speckle tracking.

After performing the bench press exercise, a slight increase in basal diameter was observed (42.13 ± 0.08 versus 48.24 ± 0.92 mm after bench press, *p* value < 0.001). The right ventricle increased the sphericity index from 0.48 to 0.65 (*p* < 0.001), increasing the diameter of the proximal and distal outflow tracts (29.43 ± 11.23 mm to 38.72 ± 15.18 mm and 28.88 ± 10.44 mm to 39.11 ± 08.55 mm, all *p* < 0.01), respectively. Right ventricular volumes also increased significantly (120.35 ± 25.31 and 168.89 ± 32.53 mL, T-student, *p* value < 0.001). All the values studied by speckle tracking showed higher values in the control group than in the baseline measurement of the weightlifters. However, after exercise, there was an increase in strain, strain rate, longitudinal and radial velocities, and their displacements. After exercise, there was a decrease in the activation delay between the basal and lateral segments in the ventricular deformity, not attributed to heart rate, and which could mean the existence of greater right ventricular synchrony, as shown in Table 3 and Table 4, Figure 3, Figure 4, Figure 5, Figure 6 and Figure 7.

**Table 3:** Diastolic function: analysis included pulsed and tissue Doppler of right ventricular filling, tricuspid annulus motion, inferior vena cava diameter, and right hepatic vein flow.

	Control Group (*n* = 60)	Before Exercise. Bench Press Group (*n* = 211)	After Exercise. Bench Press Group (*n* = 211)	*p*-Value
Right velocity E wave (m/s)	0.45 ± 0.22	0.55 ± 0.28	0.94 ± 0.12	0.001
Peak velocity A wave (m/s)	0.28 ± 0.12	0.32 ± 0.12	0.61 ± 0.32	0.001
E deceleration time (ms)	235.44 ± 56.32	202.22 ± 22.55	213.57 ± 88.32	0.001
IVRT (ms)	72.68 ± 11.34	87.24 ± 12.45	56.75 ± 17.78	0.001
Velocity right lateral E′ wave (m/s)	0.017 ± 0.15	0.021 ± 0.02	0.038 ± 0.02	0.001
Right E/E′ ratio	2.14 ± 0.28	2.61 ± 0.08	2.47 ± 0.28	N.S.
PCWP (Kuecherer equation mmHg)	12.06 ± 2.2	8.32 ± 0.12	5.60 ± 0.32	0.001
Inferior cava vein (mm) *	11.32 ± 3.36	7.08 ± 0.33	18.21 ± 3.5	0.001

IVRT Isovolumetric relaxation time (ms). PCWP: pulmonary capillary wedge pressure (mmHg). PCWP by Kuecherer equation = 35 − 0.39x systolic filling fraction of the pulmonary veins (%). * The inferior vena cava collapses in all three groups. N.S. Not significant.

**Table 4:** Global speckle tracking of right ventricle. Anova test was used.

	Control Group (*n* = 60)	Before Bench Press (*n* = 211)	After Bench Press (*n* = 211)	*p*-Value
Peak systolic longitudinal velocity (cm/s)	4.98 ± 0.12	4.01 ± 10.21	9.48 ± 3.25	0.001
peak E longitudinal velocity (cm/s)	−5.6 ± 1.35	−4.2 ± 0.87	−7.38 ± 3.15	0.0001
peak A longitudinal velocity (cm/s)	−4.01 ± 0.74	−3.75 ± 0.98	−5.14 ± 1.48	0.0001
Peak systolic radial velocities (cm/s)	4.76 ± 0.48	1.76 ± 0.48	6.17 ± 1.35	0.0001
E peak radial velocity (cm/s)	−1.75 ± 0.16	−1.45 ± 0.54	−2.21 ± 0.191	0.0001
A peak radial velocity (cm/s)	−0.11 ± 0.12	−0.09 ± 1.35	−0.6 ± 0.22	0.0001
Right ventricular longitudinal global strain (%)	−27.31 ± 1.47	−23.55 ± 2.37	−30.98 ± 2.12	0.0001
Right ventricular Longitudinal Strain delay (ms)	144.88 ± 22.52	168.92± 29.35	98.27 ± 12.11	0.0001
Right ventricular longitudinal global right Strain rate (1/s)	−1.79 ± 0.078	−1.48 ± 0.33	−2.88 ± 0.25	0.0001
Right ventricular Longitudinal systolic displacement (mm)	6.12 ± 1.18	5.09 ± 3.22	10.88 ± 1.25	0.0001
Right ventricular Radial systolic displacement (mm)	1.88 ± 1.78	1.33 ± 1.14	4.57 ± 0.96	0.0001
Right ventricular Radial displacement delay (ms)	182.14 ± 17.12	212.36 ± 18.96	124.32 ± 15.25	0.0001

### 3.5. Diastolic Function

Diastolic function parameters were normal across all groups. Baseline athletes had higher velocity right lateral E′ waves than the control group. Post-exercise, all right diastolic function parameters and pulmonary capillary wedge pressure showed improvement. Weightlifters had lower baseline speckle tracking-derived parameters for right ventricle diastolic function compared to controls, but these values significantly improved after bench press exercises, as shown in Table 3 and Table 4.

### 3.6. Ten-Year Follow-Up

In 2024, 30 weightlifters who participated in this study starting in 2014 underwent follow-up echocardiography. Their mean age was 44.23 ± 10.12 versus 42.11 ± 13.46 years in the 30 non-athletes in the control group, *p* value > 0.05. The results indicated that there were no changes in physiological values, in RVEF, right ventricular strain, or strain rate, but the S’ wave velocity decreased from 16.72 ± 0.15 to 13.12 ± 0.88 cm/s. (*p* value < 0.034) and IVA decreased from 4.88 ± 0.58 to 3.25 ± 0.09 m/s (*p* < 0.014). The 10-year follow-up echocardiography showed no significant differences in the contractility of the apical, middle, and basal segments, as shown in Table 5. This 10-year follow-up group significantly increased their echocardiographic values after exercise, as shown in Table 5. No athlete presented right ventricular hypertrophy (right ventricular wall thickness in the subcostal window was 5.21 ± 0.14 cm), ventricular dilatation, or pathological right ventricular remodeling.

**Table 5:** Ten-year follow-up of 30 athletes maintaining the same training routine. NS. the value of the *p* wave is not significant.

	Control Group (*n* = 30)	2014 (*n* = 30)	2024 Before Bench Press (*n* = 30)	2024 After Bench Press (*n* = 30)	*p*-Value
Systolic blood pressure (mmHg)	126.48 ± 11.107	120.36 ± 07.13	117.03 ± 10.21	139.22 ± 15.37	NS
Diastolic blood pressure (mmHg)	84.11 ± 12.04	78.11 ± 02.12	72.11 ± 5.28	88.81 ± 12.26	NS
Heart rate B/min	78.11 ± 15.28	65.18 ±09.12	59.88 ± 3.27	121.53 ± 14.39	NS
Breathing frequency (B/m)	17.48.33 ± 1.28	15.33 ± 1.28	15.11 ± 1.09	27.48 ± 2.21	NS
SpO_2_	97.33 ± 0.91	97.33 ± 0.91	98.78 ± 0.57	96.45 ± 0.57	NS
3D RVEF (%)	0.63 ± 0.12	0.61 ± 0.25	0.58 ± 0.33	0.76 ± 0.21	NS
IVA (m/s^2^)	2.98 ± 1.23	4.88 ± 0.58	3.25 ± 0.87	6.37 ± 1.33	0.034
S’ wave (cm/s)	12.57 ± 1.23	16.72 ± 0.15	13.12 ± 0.18	17.28 ± 1.44	0.012
TAPSE (mm)	22.39 ± 5.28	25.27 ± 4.18	24.32 ± 3.23	28.89 ± 1.47	NS
Peak systolic longitudinal velocity (cm/s)	4.14 ± 1.27	5.18 ± 0.27	4.78 ± 1.24	111.22 ± 3.41	NS
Peak systolic radial velocities (cm/s)	4.02 ± 0.87	4.76 ± 0.48	6.87 ± 1.51	6.17 ± 1.35	NS
Right ventricular longitudinal global strain (%)	−27.87 ± 1.97	−24.28 ± 1.32	−25.31 ± 1.18	−36.48 ± 2.32	NS
Right ventricular longitudinal global right Strain rate (1/s)	−1.57 ± 1.25	−1.53 ± 0.11	−1.59 ± 0.54	−2.87 ± 0.75	NS
Right ventricular Longitudinal Strain delay (ms)	119.33 ± 32.18	107.21 ± 27.78	113.41 ± 35.16	78.22 ± 24.38	NS
Right ventricular Longitudinal systolic displacement (mm)	6.88 ± 4.51	6.11 ± 2.23	7.33 ± 4.21	12.28 ± 3.11	NS
Right ventricular Radial systolic displacement (mm)	4.31 ± 2.13	4.13 ± 1.58	3.88 ± 0.49	3.48 ± 0.87	NS
Right ventricular Radial displacement delay (ms)	167.23 ± 32.69	167.23 ± 32.69	152.72 ± 21.37	81.12 ± 11.13	NS

### 3.7. Analytical and Nutritional Data of Weightlifters

Blood count and biochemical study were normal, and no increase in troponin T levels was detected (undetectable level). In the analytical tests, we had no abnormal values, except for an increase in interleukin 6 (5.63 ± 1.29 pg/mL, for a reference value of our laboratory < 2 pg/mL). Table 6. We found the following correlations in the 67 athletes who agreed to undergo a biochemical and hemogram study. Right ventricular longitudinal strain correlated with protein level (R^2^ = 0.399, *p* value = 0.021, vitamin E and zinc levels (R^2^ = 0.378, *p* value = 0.03 and R^2^ = 0.266, *p* value = 0.014, respectively). Right ventricular strain rate correlated with protein level (R^2^ = 0.511, *p* value = 0.039), PUFA value (R^2^ = 0.397, *p* = 0.013). Right ventricular radial velocity correlated with the levels of the following vitamins: vitamin A (R^2^ = 0.283; *p* value = 0.002) vitamin B (R^2^ = 0.321, *p* = 0.001; and vitamin C (R^2^ = 0.483, *p* value = 0.002). Finally, longitudinal displacement correlated with magnesium levels (R^2^ = 0.324, *p* value = 0.036). All athletes were taking protein supplements, and 28 of these athletes were taking vitamin supplements. We found no link between elevated interleukin 6 in athletes and any other studied parameter.

## 4. Discussion

Myocardial muscle fibers can enlarge and improve their contractile capacity through hypertrophy. Strength exercise typically results in concentric hypertrophy, characterized by increased muscle mass without significant dilation of the cardiac chambers. This may lead to enhancements in both diastolic and systolic function, thus improving the heart’s efficiency in filling and pumping blood. No right ventricular hypertrophy was observed, but there was a slight increase in diameters, which was more pronounced after performing bench presses.

The impact of sports on right ventricular function remains a topic of debate [36,37,38,39,40]. Some studies have reported that athletes exhibit poor right ventricular function [1,4,5,6,7,8,9,10,11,18,21,22,23], while others have found improved right ventricular function [29,31,38,39].

Compared to the control group, there was a modest reduction in the baseline S and SR values of the athletes in our study (non-professionals). Our findings align with those reported by Dalsgaard et al. [40], showing that our athletes had a higher IVA than the non-athlete population and that infusion of 30 mL/kg saline increased the strain and strain rate but did not change IVA. This finding suggests a positive cardiac remodeling that enables muscle fibers to achieve higher tissue velocities with less deformation at rest, but with a clear contractile reserve that increases significantly after bench pressing. Similar findings were reported by Starekova et al. [41] in male soccer players and competitive male triathletes. This effect may diminish with age. In our study, the weightlifting group studied after a 10-year follow-up maintained a similar strain but with lower tissue velocity. This proposes that they maintain contractile reserve over a 10-year period, if training remains consistent. Similarly, King et al. [42] observed a reduction in strain and strain rate with an increase in IVA, which they did not attribute to myocardial damage, referring to this phenomenon as “Cream masquerades as skimmed milk” and suggesting a similar hypothesis. However, this hypothesis may be incorrect and might merely reflect a higher sensitivity of IVA compared to the strain or strain rate. Kovács A et al. [25] found in an experimental study that strain and strain rate parameters closely reflected the improvement in intrinsic contractile function induced by exercise training. These changes in volume and right ventricular geometry may provide a misleading indication of right ventricular dysfunction. Our group [29], like those in other studies [17,23,24,25], observed that, as in the left ventricle, strain increased after bench press exercises. Interestingly, we determined a decrease in basal segment contractility in baseline echocardiography, which responds appropriately after exercise. Other studies have detected a decrease in basal strain and strain rate in athletes [4,43], suggesting this could be an adaptive compensatory remodeling mechanism or a reserve contractile capacity. This may also indicate athlete-specific remodeling and could help distinguish it from pathological remodeling [44].

Multiple studies, particularly those involving soldiers engaged in strenuous running, have identified right ventricular dysfunction in individuals undergoing intense exercise. This is accompanied by elevated levels of troponin I and brain natriuretic peptide, indicating a defect in contractility due to exercise-induced injury. These findings align with observations previously reported by other research groups [17,30,40]. Neilan et al. [9,10] and Wang et al. [45] identified a persistent but reversible cardiac systolic dysfunction accompanied by worsening diastolic function in amateur marathon runners [6,9,10]. Nonetheless, the potential presence of exercise-induced injury does not necessarily indicate irreversibility; it may represent a readaptation mechanism that temporarily improves systolic or diastolic function [11]. These variations can be attributed to the intensity of exercise and the overcoming of pathophysiological mechanisms that might lead to pathological remodeling and potentially lethal arrhythmias [22,23]. Additionally, differing physiological responses between strength training and endurance training could result in improvements in one form while causing deterioration in the other [43,44,45,46,47,48,49].

Interestingly, we noted that patients in the control group could have better diastolic function values in both ventricles than weightlifters at baseline, and after exercising, these parameters improve considerably, coinciding with findings from other authors [11]. This is evidenced fundamentally in parameters derived from speckle tracking.

Diastolic function has been minimally studied in athletes, particularly regarding the right ventricle. This study observed a lower PCWP in athletes both before and after bench press exercise. Additionally, right speckle tracking revealed a reduction in E waves of longitudinal and radial velocities and a decrease in right strain rate values that increased after exercise. These findings may indicate changes in left diastolic function and a right diastolic reserve, which responds to exercise by altering diastolic functions of both ventricles. This study also suggests that resistance training might affect right intraventricular synchrony, potentially indicating structural alterations in the heart due to training [23]. In addition to the hemodynamic changes that occur in the athlete with load, frequency and interventricular interaction, the myocardial fibers may undergo adaptations in both their systolic and diastolic functions, as these processes are interconnected. According to Francisco Torrent-Guasp’s theory, the dilation of the myocardial fibers in both ventricles could impact biventricular systolic and diastolic function and synchrony [50,51].

The contraction of the longitudinal fibers constitutes 80% of the right ventricle’s volume. This study observed a significant increase in IVA, S, and SR following bench press exercises. These findings suggest that the ventricles of athletes engaged in this type of training may exhibit enhanced contractility and distensibility [1,24,25,29,43].

Despite the low sample volume in the follow-up of the athletes, we detected that tissue velocities and IVA decrease, probably as a response to age. However, all echocardiographic values increased after exercise in a similar way to that of the rest of the athletes, suggesting that they continue to maintain a high functional reserve. In addition, none of them had high blood pressure or pathological remodeling suggestive of sleep apnea hypopnea syndrome, findings that are debatable due to the sample size, but certainly very promising.

Collectively, the current scientific literature indicates that nutritional supplementation strategies utilizing carbohydrates, protein, and/or amino acids is of particular importance in the responses for strength and power athletes, primarily increased muscular hypertrophy and enhanced strength expression. It optimizes the performance, recovery, and health of athletes, maintaining energy levels, repairing tissues, and preventing injuries [52]. These nutritional contributions could modify the striated muscle structure and should have an impact on the cardiac structure [53,54,55].

However, with classic echocardiography, these possible changes are imperceptible. Interestingly, we obtained a good correlation between right ventricular strain with proteins and especially with vitamin A and zinc. Similarly, we obtained a good correlation between vitamin B1, B3, and B6 levels and right ventricular radial velocity. This correlation with vitamins can be explained by their function of converting carbohydrates into energy; however, they must have other properties that are not well known, such as protection against heart failure [56,57]. Although no athlete showed elevated troponins, an increase in interleukin was observed, which may suggest that after intense exercise an inflammatory response is produced. Similar findings have been reported [58], even with modulation of the immune system [59].

### Limitations

This pilot study, with a small sample size, indicates the potential for testing our hypothesis. Conducted in a gym setting, the results need further confirmation. The participants were Caucasian men not using steroids. Despite age variability and potential biases related to sex, steroid use, or ethnicity, the speckle tracking technique remains unaffected by these factors. We believe this age group more accurately represents the general population compared to studies of young elite athletes.

The limitations of the present study also include the lack of knowledge of the fat mass of the athletes, the low sample size of the nutritional study, and the low number of controls. Nevertheless, we only used the controls to determine the normal echocardiographic values in our population.

Although the sample size of healthy individuals was small, this study indicates that exercise might improve strain and quality of life for patients with cardiac issues. Confirmation through a clinical trial is necessary. Speckle tracking measurements and decreased contractility in basal segments could help distinguish athletic cardiac remodeling from other cardiomyopathies such as arrhythmogenic right ventricular dysplasia and track ventricular remodeling in athletes. Additionally, speckle tracking may be used to investigate cardiac responses to various nutritional statuses and their effects on athletic performance [58,59,60].

## 5. Conclusions

Following bench press exercises, both systolic and diastolic functions showed improvement. Speckle tracking demonstrated a correlation with nutritional status.

## Figures and Tables

**Table 6:** Analysis performed on weightlifters.

Parameter	Result	Reference Range
Red blood cells (count)	4.93 ± 02.12 × 10^6^/μL	3.50–5.00
Hemoglobin	14.9 ± 02.21 g/dL	12.0–16.0
Hematocrit	48.7 ± 3.15%	36.0–48.0
Mean corpuscular volume	96.7 ± 09.47 fL	82.0–95.0
Mean corpuscular hemoglobin	30.3 ± 09.31 pg	27.0–32.0
Mean corpuscular hemoglobin concentration	33.4 ± 2.21 g/dL	32.0–36.0
Red blood cell distribution width (volume)	13.0 ± 01.37%	11.0–16.0
Platelets (count)	271 ± 45.38 × 10^3^/μL	130–450
Mean platelet volume	8.2 ± 0.37 fL	7.0–11.0
Leukocytes (count)	8.23 ± 0.41 × 10^3^/μL	3.90–11.70
Neutrophils (percentage)	61.80 ± 3.27%	40.00–75.00
Lymphocytes (percentage)	28.90 ± 01.49%	20.00–45.00
Monocytes (percentage)	7.50 ± 1.19%	2.00–10.00
Eosinophils (percentage)	1.60 ± 0.07%	0.00–6.00
Basophils (percentage)	0.20 ± 0.02%	0.00–1.00
Neutrophils (count)	5.09 ± 0.13 × 10^3^/μL	1.50–8.00
Lymphocytes (count)	2.38 ± 00.27 × 10^3^/μL	1.00–4.00
Monocytes (count)	0.62 ± 0.15 × 10^3^/μL	0.00–1.00
Eosinophils (count)	0.13 ± 0.01 × 10^3^/μL	0.00–0.30
Basophils (count)	0.02 ± 0.01 × 10^3^/μL	0.00–0.10
PUFA	9.8 ± 0.34 g/24 h	3–6
Glucose	73.3 ± 08.45 mg/dL	70–99
Urea	21.57 ± 03.27 mg/dL	10–50
Creatinine	0.82 ± 0.02 mg/dL	0.50–0.90
Uric Acid	3.50 ± 0.15 mg/dL	2.4–5.7
Glucose	73.28 ± 1.33 mg/dL	70–99
Lactate Dehydrogenase	157.15 ± 25.48 U/L	0–250
Sodium	143.09 ± 13.37 mEq/L	136–145
Potassium	4.41 ± 0.15 mEq/L	3.5–5.1
Creatine kinase	60.21 ± 1.32 U/L	26–192
Alkaline phosphatase	49.48 ± 2.28 U/L	35–104
Aspartate transaminase	13.74 ± 02.116 U/L	0–32
Alanine transaminase	9.32 ± 1.16 U/L	0–33
Gamma glutamyl transferase	10.57 ± 0.37 U/L	5–36
Calcium	9.71 ± 0.41 mg/dL	8.5–10.2
Phosphorus	3.62 ± 0.41 mg/dL	2.7–4.5
Iron	142.88 ± 1.49 μg/dL	37–145
Phosphorus	4.21 ± 0.38 mg/dL	2.7–4.5
Magnesium	3.01 ± 0.27 mg/dL	1.70–2.55
Copper	112.17 ± 0.61 μg/dL	70–140
Selenium	103.23 ± 0.32 mcg/L	60–120
PUFA	9.81 ± 2.62 g/24 h	3–6
Total proteins	7.92 ± 3.24 g/dL	6.4–8.3
Albumin	6.84 ± 0.23 g/dL	4–44
Cholesterol	155 ± 15.61 mg/dL	00–200
Triglycerides	63 ± 1.47 mg/dL	30–150
Thyrotropin	0.97 ± 0.18 μUI/mL	0.27–4.20
Thyroxine (free)	1.38 ± 0.03 ng/dL	0.93–1.90
Vitamin B1	6.60 ± 0.12 mcg/dL	2.8–8.5
Vitamin B2	269.0 ± 0.74 ng/mL	125–300
Vitamin B6	36.87 ± 1.27 ng/mL	5.68–42.60
Vitamin B12	469 ± 10.37 pg/mL	197–771
Vitamin C	1.07 ± 0.02 mg/L	4–20
Vitamin A	54.21 ± 0.03 μg/dL	30–100
Vitamin D (25 OH)	24.7 ± 0.10 ng/mL	20–100
Vitamin E	11.32 ± 0.14 μg/mL	5–20
Vitamin K1	0.59 ± 0.02 μg/mL	0.13–1.50
Folic Acid	24.57 ± 0.03 ng/mL	3.9–26.8
Brain Natriuretic Pro-peptide	56.23 ± 2.18 pg/mL	<100
Erythrocyte Sedimentation Rate	19.33 ± 1.23 mm/h	2–10
Troponin T, High Sensitivity	10.11 ± 0.15 ng/L	0.0–14
Interleukin 6	5.63 ± 1.29 pg/mL	<2
Testosterone (free)	47.77 ± 2.27 pg/mL	15.00–50.00
Cortisol	9.31 ± 1.22 μg/dL	4.8–19.5
Aldosterone	17.24 ± 1.35 ng/dL	3–30
Renin	2.12 ± 0.42 pg/mL	2.0–26.0
Renin (activity)	0.27 ± 0.04 pg/mL	0.28–3.70

## Data Availability

Data is unavailable due to privacy or ethical restrictions, Servicio Andaluz de Salud.

## Abbreviations

The following abbreviations are used in this manuscript:

2D	Two-dimensional
3D	Three-dimensional
ASE	American society of echocardiography
DTI	Doppler tissue imaging
EDRVD	End-diastolic right ventricular diameter, evaluated in apical 4-chamber projection, measured at the base of the right ventricle
ESC	European Society of Cardiology
IVRT	Isovolumic relaxation time
IVA	Isovolumic acceleration
RM	Repetition maximal
RVEDV	Right ventricular end-diastolic volume
IPAQ	International physical activity questionnaires; https://www.juntadeandalucia.es/export/drupaljda/salud_5af95872aeaa7_cuestionario_actividad_fisica_ipaq.pdf (accessed on 1 January 2012)
LV	Left ventricle
RVEF	Right ventricular ejection fraction
RVESV	Right ventricular end diastolic volume
S	Strain
SR	Strain rate
SRS	Systolic strain rate
TAPSE	Tricuspid annular plane systolic excursion
